# Predictors of hospital-acquired adverse drug reactions: a cohort of Ugandan older adults

**DOI:** 10.1186/s12877-022-03003-9

**Published:** 2022-04-23

**Authors:** Tadele Mekuriya Yadesa, Freddy Eric Kitutu, Robert Tamukong, Paul E. Alele

**Affiliations:** 1 Department of Pharmacy, Mbarara University of Science and Technology, Mbarara, P.O.Box 1410, Uganda; 2 Department of Pharmacy, Ambo University, Ambo, Ethiopia; 3 Pharm-Biotechnology and Traditional Medicine Center, Mbarara University of Science and Technology, Mbarara, Uganda; 4 Department of Pharmacy, School of Health Schiences, Makerere University, Kampala, Uganda; 5 Sustainable Pharmaceutical Systems (SPS) unit, School of Health Schiences, Makerere University, Kampala, Uganda; 6 Department of Pharmacology and Therapeutics, Mbarara University of Science and Technology, Mbarara, Uganda

**Keywords:** Predictors, Occurrence, Hospital-acquired, Adverse drug reaction, Older adults, Uganda

## Abstract

**Background:**

Globally, it is estimated that the number of older adults will become 2 billion by 2050. The identification of the predictors of adverse drug reaction (ADR) in hospitalized older patients is crucial to the development of prediction tools and preventive strategies to mitigate the burden of ADRs. This study aimed to determine the predictors of hospital-acquired ADR occurrence among hospitalized older adults in a low-income country.

**Methods:**

We conducted a prospective cohort of older adults admitted to medical, oncology, and surgery wards at Mbarara Regional Referral Hospital (MRRH) for a consecutive 6 months where each patient was followed up daily from admission to discharge. We used Edwards and Aronson’s definition of ADR and the Naranjo ADR Causality Scale. We employed Beer’s criteria and Lexicomp to determine potentially inappropriate medications, and drug interactions, respectively. We conducted univariate and multivariable logistic regression using Statistical Package for the Social Science (SPSS) Version 23.0.

**Results:**

Out of 523 participants with median (Inter Quartile Range) age of 67 (62–76) years, 256 (48.9%) experienced at least one ADR. Independent predictors of occurrence of hospital acquired ADRs included age of 60–75 (Adjusted odds ratio (AOR) = 1.97, 95% C.I: 1.14–3.41; *p* value = 0.015) compared to > 75 years, previous ADR in 1 year (AOR = 2.43, 95% C.I: 1.42–4.17; *p* value = 0.001), potentially inappropriate medication (AOR = 4.56, 95% C.I: 2.70–7.70; *p* value< 0.001), polypharmacy (AOR = 3.29, 95% C.I: 1.98–5.46; *p* value< 0.001)), having a Charlison Comorbidity Index (CCI) ≥ 6 (AOR = 8.47, 95% C.I: 4.85–14.99; *p* value< 0.001), having heart failure (AOR = 2.83, 95% C.I: 1.34–6.02; *p* value = 0.007) or kidney disease (AOR = 1.95, 95% C.I: 1.05–3.61; *p* value = 0.034) and a hospital stay > 10 days (AOR = 3.53, 95% C.I: 1.89–6.61; *p* value< 0.001) compared to < 5 days.

**Conclusion:**

The current prevalence of ADR is higher than previously reported in high-income countries. Disease-related factors followed by medication-related factors were shown to be the most important predictors of hospital-acquired ADRs. CCI and PIM showed the strongest association with ADR. The predictors of ADRs identified in our study were generally comparable with those reported by previous studies.

**Plain language title:**

Conditions that predispose older patients to experience harmful effects from their medications while in hospital.

**Plain language summary:**

Identifying the conditions that predispose older adults to incur harmful effects of their medications helps to plan on how best to predict, take precautions and closely follow up on them and thus, to prevent these undesirable outcomes. This study aimed to identify these conditions which determine which older adults are higher risk to incur these harmful undesirable effects of medicines. Everydayduring their hospital stay, we closely followed older patients who were 60 years and above from their entry to the hospital wards until they left the hospital. We interviewed the participants, reviewed their medication files and we also examined them physically to identify any unwanted and harmful outcome from their current medications. Out of 523 participants, almost half of them experienced at least one harmful or undesired effect related to their medicine. Conditions which predisposed them to experience a harmful effect from their medicines included being in age bracket of 60–75 years, having a history of experiencing harmful outcomes from medicines in the previous 1 year, taking a medication which was listed as potentially inappropriate for older adults, taking 5 or more medications concurrently, having a lower 10 years survival chance, having heart or kidney disease and a hospital stay > 10 days.

## Introduction

An adverse drug reaction (ADR) is defined as ‘an appreciably harmful or unpleasant reaction, resulting from an intervention related to the use of a medicinal product, which predicts hazard from future administration and warrants prevention or specific treatment, or alteration of `the dosage regimen, or withdrawal of the product [1]. Pharmacovigilance studies are crucial for the identification of rare but serious ADRs [2]. A substantial increase in the older adults population is apparent over the past decades. It is estimated that the global older population will become 2 billion by 2050 [3].

The latest systematic review reported a prevalence of ADRs of 22% among hospitalized older adults; 19% for high-income countries and 29% for low-income countries [4]. In the UK, between 2007/8 and 2014/15, the emergency admissions due to ADRs increased by 53.4%, and the number of bed days that were used due to ADRs also increased by 51.5% over the same period [5].

Studies report several factors that influence the occurrence of ADRs including: older age [6–8], female gender [9–11], higher number of co-morbidities [7, 12–14], increased number of drugs [15–17], receiving potentially inappropriate medication (PIM) [6, 7, 13], use of herbal remedy in previous 4 weeks [18, 19], renal diseases [20, 21], hepatic conditions [7, 14], having heart failure [7, 14, 22], higher CCI [7, 13, 14] [23], previous ADRs [24] and type of health care setting [13]. Renal failure, either concealed (OR, 1.61; 95% CI, 1.15–1.25) or overt (OR, 2.02; 95% CI, 1.54–2.65) was associated with the occurrence of ADR [21].

A cohort study conducted among hospitalized general adults in Uganda identified that taking six or more conventional medicines (OR = 2.72, 95% CI 1.79 to 4.13), herbal medicine use within 4 weeks preadmission (OR = 1.68, 95% CI 1.16 to 2.43), hospitalization in previous 3 months (OR = 1.57, 95% CI 1.09 to 2.26) and admission at gynecology ward were risk factors for possible hospital-acquired ADRs [18].

Potential risk factors for ADRs in low-income countries (LICs) differ from high-income countries (HICs). For LICs, they include greater proportions of patients taking antituberculosis (anti-TB) and antiretroviral therapy (ART), a high prevalence of anemia and malnutrition, the highly prevalent use of traditional medicines, higher incidence of concomitant anti-TB drugs, and ART with overlapping adverse effects [25].

The determination of the predictors of ADR occurrence in hospitalized older patients is crucial to develop prediction tools and preventive strategies which help to mitigate the burden of ADRs in clinical as well as economic aspects in the LICs [26, 27].

However, studies that report predictors of ADR occurrence among the older population are scarce in low and middle-income countries (LMICs) [28]. Yet the documented increase in life expectancy and the proportion of the older population with the attendant polypharmacy and comorbidities is expected to increase in-hospital ADR incidence among this age group. Understanding the predictors of the occurrence of ADRs among the older given their inherent higher risk is imperative to any prevention strategies, reduction of morbidity, and improvement of patient safety. The current study, therefore, aimed to determine the predictors of hospital-acquired ADR occurrence among hospitalized older adults in a low-income country.

## Methods

### Study setting and period

The current study was conducted at MRRH, the largest public referral hospital in southwestern Uganda with a 600-bed capacity. The hospital consists of the Emergency and Critical Care ward, Oncology, Medicine, Surgery, Gynecology and Obstetrics, Psychiatry, Pediatrics and TB ward in addition to several outpatient clinics. This cohort study was conducted from the 9th of November, 2020 to the 7th of May, 2021.

### Study design

A prospective cohort study was conducted.

### Study population

We included all inpatients 60 years and older that were admitted to Medical, Oncology, and Surgery wards of MRRH during the study period who gave their informed consent to participate in the study. We excluded patients who died or were discharged within 48 h and those with any level of unconsciousness or coma.

### Sample size and sampling technique

#### Sample size determination

Sample sizes required to test each of the potential risk factors were determined using EPI INFO™ Version 7.2.3.1 using the Kelsey formula at a confidence level of 95% and power of 80%. Based on our findings from the previous systematic review, we identified all the potential risk factors of ADRs among hospitalized older patients. We included all the clinically important variables in the current cohort: gender, age, polypharmacy, CCI, PIM, cancer, heart failure, liver disease, renal disease, medication history (3 months), allergy history, previous ADR (1 year), use of an herbal drug in previous 1 month and duration of hospital stay. The minimum sample sizes required for each of the included variables ranged from 84 (42 patients with herbal drug use and 42 without herbal drug use in the previous 1 month) for previous use of an herbal drug to 500 (250 patients with polypharmacy and 250 without it during admission). Thus, a minimum target sample size of 500 was considered adequate for our study. Adding 10% for possible non-response, incomplete patient files, too early discharge of the patients, the target sample size of 556 older adults of 60 years and older were required to test the predictors of ADR. Post-study calculation of sample size revealed that we achieved higher power ranging from 88 to 99% for most of the important variables including age, polypharmacy, CCI, PIM, having heart failure, liver disease, renal disease or cancer, allergy history, previous hospitalization, clinically significant drug-drug interaction (DDI), and history of ADR. A lower power of 74% for gender and 51% for previous use of herbal drugs than anticipated was observed.

#### Sampling techniques

From patient records of the corresponding period of 6 months of the previous year (2019) over 1150 older patients were admitted to Medical, Oncology, and Surgery wards of MRRH. Using this estimate, we determined that enrolling half of all the admissions through random selection using random numbers generated in MS Excel 2019 would achieve the target sample size of 556 patients in 6 months.

### Study variables

The authors conducted a systematic review on predictors of ADRs in hospitalized older adults and the results are published elsewhere [4]. We included 15 predictors reported by previous studies as independent variables in our observational study. These included: older age [6–8], female gender [9–11], having liver disease [7, 14], heart failure [7, 14, 22], renal disease [6, 7, 13, 14, 29], cancer [22], Diabetes [22], or higher CCI [7, 13, 14], previous ADRs [24], type of health care setting [13], length of hospital stay [13, 30–32], emergency admission [30], polypharmacy [6, 7, 11, 13, 14, 19, 29–32], complementary or alternative medicine use [19], receiving PIM [6, 7, 13]. However, we excluded previously reported predictors such as previous falls, atrial fibrillation, dementia and smoking which are rare in the current study’s setting because adequate power could not be achieved during a 6-months study period. On the other hand, we included four more potential predictors of hospital-acquired ADRs which are not reported in published literature including allergy history, hospital admission in the previous 3 months, and medication use in previous 3 months, and clinically significant drug-drug interactions. Thus, we examined a total of 19 variables for prediction of ADR occurrence which makes the current study more comprehensive than previous similar studies. The outcome variable of the current cohort study was the occurrence of at least one new ADR at any time between admission and discharge dates.

### Data collection

Data were collected by a research team consisting of four research assistants; two physicians in the final year of their master of medicine in internal medicine and two pharmacists. First, we used a pretested questionnaire to collect patient information on socio-demographic characteristics, medical and medication history, previous adverse drug events, drug allergies, use of over-the-counter and herbal medicines. Then, we reviewed patients’ medical records for working diagnosis, previous allergies, and clinical and laboratory data within 48 h of admission. Every day during their hospital stay, patients were interviewed and their information was updated. Lastly, the team’s physicians conducted physical assessments and interpreted clinical, laboratory, and diagnostic data relevant to ADR monitoring and identification on a daily basis.

The Beers Criteria [33] was used to identify PIMs. Polypharmacy was defined as the concurrent use of five or more drugs (active pharmaceutical ingredients). Lexicomp® available on© 2021 UpToDate was used to detect clinically significant drug-drug interactions. The medications suspected for ADRs were classified according to the WHO-Anatomical Therapeutic Chemical (ATC) classification [34]. Charlson Comorbidity Index was used to grade the complexity of comorbidities [35].

### Procedures to identify ADRs

We adopted Edwards and Aronson’s definition of ADRs [1]. ADRs were first suspected when there was a relationship between the time of drug administration and the onset and course of the adverse reaction while excluding other potential causes [36]. The known ADR profile of each drug was evaluated based on British National Formulary [37] and UpToDate [38]. All ADRs suspected by the principal investigator were discussed and agreed on with the team of experts consisting of the principal investigator (senior clinical pharmacist), another senior pharmacist, and a senior physician. The rating of the causal relationship of an ADR and the suspected medication was done using the Naranjo ADR assessment scale [39], and all possible, probable and definite ADRs were considered. The definition of specific ADRs as well as their severity and clinical outcomes were published elsewhere [40].

First, we screened all the enrolled patients for community-acquired ADRs immediately after admission before newly prescribed medications were administered. These ADRs were excluded from hospital-acquired ADRs and recorded as a part of the previous ADRs.

Second, the causal relationship of an ADR with the suspected drug was conducted by applying the Naranjo ADR assessment scale [39]. We included all ADRs rated as possible, probable or definite whereas we excluded those rated as doubtful. Thirdly, the team of experts consisting of the principal investigator, another senior pharmacist, and a senior physician, met daily to review, discuss and reach consensus on the suspected ADRs and a majority decision of atleast two of the three members was applied otherwise.

### Data analysis and interpretation

The data were entered and cleaned by EpiInfo version 7.2.3.1 and then transferred to and analyzed by IBM Statistical Package for the Social Sciences (SPSS version 23.0 Inc., Chicago, Illinois). Cross-tabulation was made to determine the number of patients with each independent variable who had encountered at least one ADR and those without any ADRs. Univariate analysis and multivariable logistic regression were employed to determine the predictors of ADRs. The chi-square test was used to compare the characteristics of patients with and without ADRs. Independent variables with *p* value*<* 0.25 in univariate logistic regression and with VIF < 3 during multicollinearity test were included for the multivariable logistic regression model. First, we employed the backward conditional method of logistic regression that is recommended for cohort studies when participants with exposures are matched with those without the exposure with a given ratio [41]. As explained by previous derivational studies [7, 31], we then applied the backward conditional logistic regression to evaluate for any important difference. Both methods similarly showed 8 out of the 13 independent variables to be significantly associated with the occurrence of hospital-acquired ADRs. At each step, variables were removed from the next step when the *p*-value ≥0.1 for each category of the variables. Thus, we identified all the independent variables with *p* values < 0.05 at the last step of the multivariable logistic regression as the independent predictors of hospital-acquired ADRs in older adults. Moreover, we did the multivariable logistic regression of all the 19 independent variables to confirm if the removal of the clinically important variables at the univariate level had considerably affected the final model. However, the same 8 variables reached the final model and no change in odds ratios of the independent predictors was observed. A *p*-value of *<* 0.05 was considered statistically significant in all analyses.

### Ethical considerations

This study was conducted in accordance with the Declaration of Helsinki [42]. Approval to conduct the study was obtained from Mbarara University Institutional Research Ethics Committee (Reference No: MUREC 1/7) and Uganda National Council for Science and Technology (Reference No: HS992ES).

## Results

### Participants’ characteristics

A total of 523 older adults aged 60 to 103 years and with a median (Inter Quartile Range) age of 67 (62-76) years, admitted to the Medical, Oncology, and Surgery wards of MRRH between the 9^th^ of November 2020 to the 7^th^ of May 2021, were studied. Each patient was enrolled on the first day of admission to the wards and monitored once daily until discharge except on Sundays. A total of 269 (51.4%) of them were males and the median age of the participants was 67(62-76) years. The duration of hospital stay ranged from 2 to 34 days with a median follow-up duration of 8.9 days with an IQR of 4-12 days. The time from ward admission to the detection of the first ADR ranged from one to 28 days with a median of 10 days and IQR of between 6 and 14 days.

A total of 326 (62.3%) of the participants were admitted through the Emergency department whereas 262 (50.1%) of them were admitted to the Medical ward. Similarly, 272 (52%) patients reported the use of the herbal product in the previous 4 weeks. A total of 152 (29.1%) had a previous ADR and 39 (7.5%) reported a history of allergy. Polypharmacy, clinically significant drug interactions, and PIMs were incurred by 278 (53.2%), 223 (42.6%), and 219 (41.9%) of the patients. The majority (338, 64.6%) had at least one comorbid condition; 115 (22.0%) had renal disease, 81 (15.5%) had a liver disease and 184 (35.2) had confirmed cancer whereas 190 (36.3%) were rated with a CCI of >=6. Lastly, 157 (30.0%) stayed in the hospital for greater or equal to 11 days. The univariate analysis showed that the prevalence of ADR was higher among patients with previous ADR (68.4%), history of allergy (69.2%), PIM (75.8%), polypharmacy (72.7%), clinically significant drug-drug interaction (65.5%), CCI of ≥6 (85.8%), renal disease (70.4%), liver disease (72.8%), heart failure (73.0%) and a hospital stay of ≥11 days (63.7%) (Table 1).

**Table 1:** Univariate analysis for patient, disease, and drug characteristics of older adults admitted at MRRH, southwestern Uganda from November 2020 to May 2021

Exposures		Frequency (%) (***N*** = 523)	Outcome	
Variable	Categories		‘No ADR’ Frequency (%)	ADR Frequency (%)
Gender	Female	254 (48.6)	118 (46.5)	136 (53.5)
	Male	269 (51.4)	149 (55.4)	120 (44.6)
Age in years; median (Interquartile Range) = 67 (62–76)	60–75	356 (68.1)	175 (49.2)	181 (50.8)
	> = 75	167 (31.9)	92 (55.1)	75 (44.9)
Ward of admission	Medical	262 (50.1)	123 (46.9)	139 (53.1)
	Surgery	163 (31.2)	100 (61.3)	63 (38.7)
	Oncology	98 (18.7)	44 (44.9)	54 (55.1)
Type of admission	Not Emergency	197 (37.7)	113 (57.4)	84 (42.6)
	Emergency	326 (62.3)	154 (47.2)	172 (52.8)
Previous admission (3 months)	No	293 (56.0)	145 (49.5)	148 (50.5)
	Yes	230 (44.0)	122 (53.0)	108 (47.0)
Previous ADR in 1 year	No	371 (70.9)	219 (59.0)	152 (41.0)
	Yes	152 (29.1)	48 (31.6)	104 (68.4)
Allergy history	No	482 (92.5)	254 (52.7)	228 (47.3)
	Yes	39 (7.5)	12 (30.8)	27 (69.2)
Medication use in previous 3 months	No	49 (9.4)	25 (51.0)	24 (49.0)
	Yes	474 (90.6)	242 (51.1)	232 (48.9)
Previous use of the herbal drug in 4 weeks	No	251 (48.0)	124 (49.4)	127 (50.6)
	Yes	272 (52.0)	143 (52.6)	129 (47.4)
PIM^a^	No	304 (58.1)	214 (70.6)	90 (29.6)
	Yes	219 (41.9)	53 (24.2)	166 (75.8)
Polypharmacy^b^	No	245 (46.8)	191 (78.0)	54 (22.0)
	Yes	278 (53.2)	76 (27.3)	202 (72.7)
Clinically significant DDI^c^	No	300 (57.4)	190 (63.3)	110 (36.7)
	Yes	223 (42.6)	77 (34.5)	146 (65.5)
Comorbidity	No	185 (35.4)	135 (73.0)	50 (27.0)
	Yes	338 (64.6)	132 (39.1)	206 (60.9)
CCI category^d^	<=5	333 (63.7)	240 (72.1)	93 (27.9)
	> = 6	190 (36.3)	27 (14.2)	163 (85.8)
Kidney disease^e^	No	408 (78.0)	233 (57.1)	175 (42.9)
	Yes	115 (22.0)	34 (29.6)	81 (70.4)
Liver disease	No	442 (84.5)	245 (55.4)	197 (44.6)
	Yes	81 (15.5)	22 (27.2)	59 (72.8)
Heart failure	No	449 (85.9)	247 (55.0)	202 (45.0)
	Yes	74 (14.1)	20 (27.0)	54 (73.0)
Diabetes	No	460 (88.0)	238 (51.7)	222 (48.3)
	Yes	63 (12.0)	29 (46.0)	34 (54.0)
Cancer	No	339 (64.8)	181 (53.4)	158 (46.6)
	Yes	184 (35.2)	86 (46.7)	98 (53.3)
Length of Hospital stay	<=5	180 (34.4)	110 (61.1)	70 (38.9)
	5–10	186 (35.6)	100 (53.8)	86 (46.2)
	> = 11	157 (30.0)	57 (36.3)	100 (63.7)

*ADR* Adverse drug reaction

^a^*PIM* Potentially inappropriate medication using 2020 Beer’s criteria

^b^Polypharmacy: The use of five or more different active ingredients of medicines

^c^DDI: Clinically significant drug-drug interaction using Lexi Comp

^d^CCI: Charlison Comorbidity index for 10 years survival

^e^Kidney disease: any documented structural renal condition or eGFR< 90 mL/min/1.73m^2^

### Adverse drug reactions

Out of 523 participants, 256 experienced at least one ADR; giving an incidenceof 48.9% (95% C.I: 44.6, 53.2%). Overall, 365 ADRs were detected during 4702 person-days of follow-up.

### Predictors of adverse drug reactions

Among 19 independent variables that were tested for association at univariate logistic regression, 13 showed statistical significance including: female gender (*p* value = 0.041), age of 60–75 (*p* value = 0.041), medical ward compared to surgery (*p* value = 0.004), emergency admission (p value = 0.025), having history of drug allergy (*p* value = 0.010), previous ADR (*p* value< 0.001), clinically significant drug interaction (*p* value< 0.001), PIM (*p* value< 0.001), polypharmacy (*p* value< 0.001), CCI (*p* value< 0.001), having kidney disease (*p* value< 0.001), heart failure or liver disease (*p* value< 0.001) as well as hospital stay of 11 days or longer (*p* value< 0.001) were all significantly associated with hospital-acquired ADRs (Table 2). Four variables including admission to hospital in the previous 3 months, history of medication use in previous 3 months, use of an herbal drug in previous 4 weeks, and having diabetes were excluded from multivariable analysis because each showed a *p*-value > 0.25. Thus, we included all the 13 variables that were statistically significant at the univariate logistic regression level and 2 more clinically important variables (age category and diagnosis with cancer) that were not statistically significant but with *p* values< 0.25). The age category [6–8] and diagnosis with cancer [22] were previously shown to be independent predictors of ADRs among hospitalized older adults. No significant multicollinearity was observed between any of the independent variables; the highest VIF being 2.722.

**Table 2:** Univariate and multivariable logistic regression for the risk factors of ADRs among hospitalized older adults, MRRH, Uganda

Exposure variables		COR (95% C.I)	***P*** value	AOR (95% C.I)	***P*** value
Variables	Categories				
Gender	Female	1.43 (1.02–2.02)	**.041**	1.39 (0.61–1.71)	0.932
	Male	1.00		1.00	
Age in years	60–75	1.27 (0.88–1.84)	0.206	1.97 (1.14–3.41)	**0.015**
	> = 75	1.00		1.00	
Ward	Medical	1.00		1.00	
	Surgery	0.56 (0.38–0.83)	**0.004**	0.64 (0.35–1.19)	0.158
	Oncology	1.09 (0.68–1.73)	0.729	1.40 (0.59–3.33)	0.442
Type of admission	Not Emergency	1.00		1.00	
	Emergency	1.50 (1.05–2.15)	**0.025**	1.36 (0.66–2.79)	0.400
Previous admission in 3 months^*^	No	1.00		^*^	^*^
	Yes	0.87 (0.61–1.23)	0.420	^*^	^*^
Previous ADR in 1 year	No	1.00		1.00	
	Yes	3.12 (2.10–4.66)	**< 0.001**	2.43 (1.42–4.17)	**0.001**
Allergy history	No	1.00		1.00	
	Yes	2.51 (1.24–5.06)	**0.010**	1.15 (0.40–3.27)	0.800
Medication use in previous 3 months^*^	No	1.00		^*^	^*^
	Yes	1.00 (0.55–1.80)	0.996	^*^	^*^
Previous use of an herbal drug in 4 weeks^*^	No	1.00		^*^	^*^
	Yes	0.88 (0.63–1.24)	0.469	^*^	^*^
PIM^a^	No	1.00		1.00	
	Yes	7.45 (5.02–11.06)	**< 0.001**	4.56 (2.70–7.70)	**< 0.001**
Polypharmacy	No	1.00		1.00	
	Yes	9.40 (6.30–14.04)	**< 0.001**	3.29 (1.98–5.46)	**< 0.001**
Clinically significant DDI^b^	No	1.00		1.00	
	Yes	3.28 (2.28–4.71)	**< 0.001**	0.68 (0.37–1.23)	0.201
CCI^c^	<=5	1.00		1.00	
	> = 6	10.69 (6.89–16.58)	**< 0.001**	8.47 (4.85–14.99)	**< 0.001**
Kidney disease	No	1.00		1.00	
	Yes	3.17 (2.03–4.95)	**< 0.001**	1.95 (1.05–3.61)	0.034
Liver disease	No	1.00		1.00	
	Yes	3.34 (1.97–5.63)	**< 0.001**	1.96 (0.95–4.05)	0.70
Heart failure	No	1.00		1.00	
	Yes	3.30 (1.91–5.70)	**< 0.001**	2.83 (1.34–6.02)	**0.007**
Diabetes^*^	No	1.00		^*^	^*^
	Yes	1.26 (0.74–2.13)	0.396	^*^	^*^
Cancer	No	1.00		1.00	
	Yes	1.31 (0.91–1.87)	0.146	0.88 (0.42–1.84)	0.730
Hospital stay (days)	<=5	1.00		1.00	
	5–10	1.35 (0.89–2.05)	0.156	1.64 (0.90–3.00)	0.106
	> = 11	2.76 (1.77–4.29)	**< 0.001**	3.53 (1.89–6.61)	**< 0.001**

*C.I* Confidence Interval, *COR* Crude Odds Ratio, *AOR* Adjusted Odds Ratio

^a^*PIM* Potentially inappropriate medication

^b^*DDI* Drug-drug interaction

^c^*CCI* Charlison Comorbidity inde

^*^Excluded from multivariable logistic regression at univariate level; Bold: Statistically significant

Accordingly, seven variables including gender, ward, type of admission, allergy history, DDI, having liver disease and having cancer showed no significant association with the occurrence of at least one ADR. Eight independent variables that maintained their significance during multivariable logistic regression were identified as the predictors of ADRs in hospitalized older adults. These included: age of 60–75 (AOR = 1.97, 95% C.I: 1.14–3.41; *p* value = 0.015) compared to > 75 years, previous ADR in 1 year (AOR = 2.43, 95% C.I: 1.42–4.17; *p* value = 0.001), PIM (AOR = 4.56, 95% C.I: 2.70–7.70; *p* value< 0.001), polypharmacy (AOR = 3.29, 95% C.I: 1.98–5.46; *p* value< 0.001)), having CCI ≥ 6 (AOR = 8.47, 95% C.I: 4.85–14.99; *p* value< 0.001), having heart failure (AOR = 2.83, 95% C.I: 1.34–6.02; *p* value = 0.007) or kidney disease (AOR = 1.95, 95% C.I: 1.05–3.61; *p* value = 0.034) and a hospital stay > 10 days (AOR = 3.53, 95% C.I: 1.89–6.61; *p* value< 0.001) compared to < 5 days (Table 2).

Patients aged 60 to 75 years of age had 1.97 times higher odds of experiencing an ADR during their hospital stay as compared to those older than 75 years. Patients that had experienced ADR in the previous year had 2.43 times higher odds of getting an ADR during the current admission. Patients who took a PIM had 4.56 times higher odds of experiencing a hospital-acquired ADR compared to those without a PIM and patients on polypharmacy had 3.29 times higher odds of experiencing in-hospital ADR compared to those without. Patients with heart failure and those that stayed in the hospital for longer than 10 days had 3.04 and 3.62 times higher odds of experiencing an ADR than those without ADR and those with a hospital stay duration of 5 days or less, respectively.

## Discussion

During this cohort study, an incidence of ADR in hospitalized older patients was determined to be 48.9% Eight independent variables including 60–75 years of age as compared to older than 75 years, an ADR history in previous 1 year, taking PIM, being on polypharmacy, having CCI ≥ 6, having heart failure or kidney disease and a hospital stay > 10 days compared to < 5 days were identified to be independent risk factors of ADRs.

The current incidence of ADR in older inpatients is considerably higher than prevalences previously reported from high-income countries: 6% in Canada [22], 6.5% in Italy [14], 13% in the UK [32], 15% in Japan [30], and 26% in Ireland [6]. This may be explained by absolute lack of active clinical pharmacovigilance in our setting. Studies in Uganda showed lack of capacity to monitor medicines and evaluate risks as well as under-reporting of ADRs [43, 44].

On the otherhand, the predictors of ADRs identified in our study are generally comparable with those reported by previous studies from middle and high income countries. Our study showed that disease-related factors followed by medication-related factors to be the most important predictors of hospital-acquired ADRs. Most previous studies on ADRs also confirmed that disease and drug characteristics, rather than the study setting, determines the risk of in-hospital ADRs among older patients [6, 7, 13].

The younger population aged 60 to 75 years had almost twice higher odds of experiencing ADRs during their hospital stay than those older than 75 years of age. This is comparable with other studies of older adults with lower median age (< 75 years) that reported higher ADR prevalence [8, 13, 45–47]. A study in Belgium and another one in the UK showed that very old age inpatients experienced the same or lower incidence of ADRs compared to other older adults. A possible explanation was that the majority of them suffered from cognitive impairment or inability to effectively communicate the symptoms commonly associated with an ADR [11, 32]. In the current study setting, compared to the very old, older adults in their 60s and 70s tended to present with more severe and complex chronic comorbidities whereas the very old patients (> 80 years) were hospitalized mainly because of acute conditions like pneumonia, malaria, falls, or gastroenteritis with or without comorbidity. Thus, the latter may have had less complex comorbidity and thus, fewer medications, resulting in a lower prevalence of ADR. In line with this argument, in the current study, the mean number of medications used in those 60–75 years was higher; 5.4 ± 2.8 compared to 4.9 ± 2.4 in those aged 75 years and older.

On the other hand, a study showed that patients with ADRs were significantly older [6]. This deviation from the general trend described above is likely due to that study’s confounders including the complexity and number of comorbid conditions, the classes, and the number of medications used.

Patients that had experienced ADR in the previous year showed more than twice higher odds of suffering ADRs during the current admission than those who had not. This was consistent with previous studies [14, 29, 48]. There are three arguments in support of this observation. First, if the previous ADR is a hypersensitivity reaction, re-exposure to the same or related medication can induce another reaction. Secondly, type A ADRs that occurred previously tend to be incurred more likely as the patient is given progressively higher doses or simply the probability of incurring ADR will increase as the patient is exposed to that medication repeatedly. Lastly, the prior ADR can directly result in long-term effects that reduce the threshold of future ADRs. This highlights the importance of comprehensive scrutiny to medication history, allergy history, and previous ADR during admission of older patients.

Beer’s criteria were intended for patients 65 years and older, but the evidence was made from studies with older adults with the mean or median age of participants older than 65 years [33]. In the current study, the median age was 67 and thus we operationalized PIM as the application of the 2019 Beer’s criteria for all patients 60 years and older. Applying Beer’s criteria [33], patients that took at least one PIM were about 4.56 times more likely to have a hospital-acquired ADR compared to those without a PIM. Other studies in hospitalized older adults that employed STOPP criteria also identified PIM as an independent risk factor of ADR [6, 7]. PIMs are medications that are typically best avoided by older adults in most circumstances or under specific situations, such as in certain diseases or conditions. Identifying and avoiding medications from this list substantially reduces the burden of ADR in the older population [33]. To this effect, the application of the Screening Tool of Older Persons’ Prescriptions (STOPP) and The Norwegian General Practice criteria (NORGEP) to hospitalized older patients demonstrated effectiveness in avoidance of the use of PIMs and thus a reduction in risk of ADRs [49, 50].

Patients on polypharmacy (concurrent use of 5 or more medicines) had significantly higher odds of experiencing a hospital-acquired ADR compared to their controls. This is in line with many studies in hospitalized older patients that revealed polypharmacy to be an independent risk factor for ADR [6, 7, 11, 13, 14, 19, 29–32]. Each medication carries a risk of ADR at doses normally used by humans. Thus, as the number of concurrent medications increases, the risk of experiencing an ADR will also rise. Moreover, more medications may increase the probability of drug-drug interaction that, in turn, can be associated with dose-related ADRs known as Type A ADRs [51]. Though many older adults need multiple concurrent medications to manage their comorbid conditions, avoiding or stopping unnecessary drugs including those initiated without a definite diagnosis, those ordered to treat avoidable ADRs of other drugs should be actively done. Likewise, drugs ordered to treat conditions that can be better managed with non-pharmacologic therapies or lifestyle modifications should be stopped [52]. Prescribers and pharmacists should work together with patients and caretakers to minimize avoidable polypharmacy in hospitals as well as in the community, to prevent ADRs and to optimize patient outcomes in the older adults.

Having a CCI ≥ 6 was independently associated with hospital-acquired ADRs among hospitalized older adults. A higher CCI is related to more complexity or number of comorbidities and lower 10-year survival. Comorbidity usually requires concurrent use of multiple drugs, and thus the risk of experiencing an ADR may rise. Previous studies also showed that more comorbidities and a higher CCI were significant predictors of ADRs [7, 13, 14]. Clinical pharmacovigilance systems need to give a special emphasis on preventing and monitoring ADRs in this group of patients. However, ADRs are more difficult to detect in older populations with multiple comorbidities [53]. Thus, clinicians should always consider ADR high in the differential diagnosis of clinical symptoms [54].

We identified having heart failure as another disease-related independent predictor of ADR. Three previous studies [7, 14, 22] in this group of patients also identified heart failure as a predictor of ADRs. This can be explained by multiple concurrent medications in heart failure patients and the high risk of ADR associated with cardiovascular drugs [55]. A large previous cohort revealed that cardiovascular drugs are independently associated with ADRs [22]. A more judicious prescription, closer monitoring, and slower titration of cardiovascular drugs is warranted in older adults.

Having kidney disease was also shown to be a disease-related predictor of ADR. Several previous studies in older patients have also reported having a kidney disease as a risk factor for occurence ADRs [6, 7, 13, 14, 29]. Renal impairment decreases the excretion of drugs that are mainly eliminated through the kidney. This may lead to a higher incidence of ADRs that are dose-related including type-A and C [51]. Both concealed and overt renal failure was revealed to be an important risk factor for ADR [21].

Patients that stayed in the hospital for longer than 10 days were about 3.62 times more likely to acquire an ADR as compared to those with a hospital stay duration of 5 days or less. This is in line with previous studies in this population of patients that showed longer hospital stay as a predictor of hospital-acquired ADRs [13, 30–32]. A higher number of drugs, rapid change of dosage regimens, and potential drug interactions might be experienced more often as patients stay longer in the hospital. Moreover, the use of medications for prevention or treatment of hospitalization-related complications including ADRs might result in more ADRs. Several studies identified a higher incidence of ADRs during hospitalization compared to the prevalence at admission [56, 57]. Additionally, a more acute and complex illness during hospitalization might narrow the threshold for an ADR in older adults.

The other variables of gender, ward setting, emergency of the admission, previous admission in the last 3 months, previous drug allergy, medication use in previous 3 months, previous herbal drug use, clinically significant drug interaction, having any of liver disease, diabetes, or cancer were not significantly associated with ADR among hospitalized older patients.

These findings are in contrast to several previous studies that showed female gender [7, 8, 11, 29, 47], having a liver disease [7, 14], having diabetes or cancer [22] as independent risk factors of ADR in this population of patients. This deviation is probably because of the difference between the participants. Particularly, we have noted a relatively lower median age, and a higher proportion of cancer patients not currently on chemotherapy. Moreover, a higher proportion of patients with acute infectious conditions with little chronic comorbidity might explain the insignificance of some of the variables which were shown to be important predictors in previous studies. Finally, the lack of regular monitoring of liver function tests, blood sugar level, serum electrolytes, and coagulation tests in our setting might have contributed to a lower rate of ADR detection in these body systems.

The strengths of the current study include: prospectively monitoring for the occurrence of hospital-acquired ADRs with consistent procedures and follow-ups, clear definitions of ADRs, and by employing validated tools. We included more variables than most of the previous studies in this population of patients; involving almost all of the previously reported clinically important independent variables. We also attempted to optimize the quality of ADR detection by engaging physicians and senior clinical pharmacists who independently detected and ruled out ADRs. Similarly, we managed to closely approximate the planned ratios of the number of unexposed to exposed patients for all the independent variables. On the other hand, the potential limitations of the current cohort include the unexpected exclusion of patients suspected of COVID-19, insufficient power for two variables (gender and use of herbal medicine in previous 1 year), and the exclusion of exposures that were rare in the study area and thus, could not be powered adequately during this study period (previous falls, atrial fibrillation, dementia, and smoking). Similarily, patients were monitored for ADRs only until discharge and all ADRs that occur afterwards were probably missed. Morever, the lack of assessment of functional status of the patients is another limitation of the current study. Lastly, the absence of country or region-modified versions of PIM criteria and the use of foreign based criteria may be inadequate. Thus, we would recommend that future researchers plan for a larger cohort and include more variables that could potentially confound the final model of ADR prediction in older adults.

## Conclusions

Almost half of the older adults experienced an ADR during the current hospitalization. The current incidence is remarkably higher than those reported by previous studies. About one-half of the patients were on polypharmacy whereasover 2 out of 5 were given PIM. The predictors of ADRs identified in our study are generally comparable with those reported by previous studies from middle and high income countries. Disease-related factors followed by medication-related factors were shown to be the most important predictors of hospital-acquired ADRs. Having a higher CCI and taking PIM showed the strongest association with ADR.

## Data Availability

The datasets used and analyzed during the current study are available from the corresponding author on reasonable request.

## Abbreviations

ACE: African Center of Excellence
ADR: Adverse Drug Reaction
AIDS: Acquired Immunodeficiency Syndrome
AOR: Adjusted Odd Ratio
ART: Anti-Retroviral Therapy
ATC: Anatomical Therapeutic
BUN: Blood Urea Nitrogen
CCI: Charlson Comorbidity Index
CI: Confidence Interval
DDI: Drug-Drug Interaction
COR: Crude Odd Ratio
eGFR: Estimated Glomerular Filtration Rate
HIV: Human Immunodeficiency Virus
MRRH: Mbarara Regional Referral Hospital
MUST: Mbarara University of Science and Technology
NORGEP: The Norwegian General Practice criteria
REC: Research Ethics Committee
PIM: Potentially Inappropriate Medications
SPSS: Statistical Package for the Social Science
STOPP: Screening Tool of Older Persons’ Prescriptions
TB: Tuberculosis
UK: United Kingdom
UNCST: Uganda National Council for Science and Technology
WHO: World Health Organization
VIF: Variance Inflation Factor
